# INTRODUCTION: What is Health Justice?

**DOI:** 10.1017/jme.2023.2

**Published:** 2022

**Authors:** Lindsay F. Wiley, Ruqaiijah Yearby, Brietta R. Clark, Seema Mohapatra

**Affiliations:** 1. UCLA SCHOOL OF LAW, LOS ANGELES, CA, USA; 2. THE OHIO STATE COLLEGE SCHOOL OF LAW, COLUMBUS, OH, USA; 3. LOYOLA LAW SCHOOL, LOYOLA MARYMOUNT UNIVERSITY, LOS ANGELES, CA, USA; 4. SOUTHERN METHODIST UNIVERSITY SCHOOL OF LAW, DALLAS, TX, USA.

**Keywords:** Social Justice, Community, Social Determinants of Health, Public Health, Health Care Access

## Abstract

Health justice is both a community-led movement for power building and transformational change and a community-oriented framework for health law scholarship. Health justice is distinguished by a distinctively social ethic of care that reframes the relationship between health care, public health, and the social determinants of health, and names subordination as the root cause of health inequities.

Health justice is not only a framework for scholarly discourse and research, but also a movement for power building and transformational change to eliminate health inequities and secure distinctively collective interests in access to health care and healthy living conditions. As a framework for health law and policy scholarship, health justice focuses particularly on the role of laws, policies, and institutions in creating, perpetuating, and (potentially) dismantling subordination within health care, public health, and beyond — which it names as the root cause of health inequities. As a movement, health justice seeks to recognize and build the power of individuals and communities affected by health inequities to create and sustain conditions that support health and justice.

## Health Justice as a Scholarly Framework

As a framework for advocating for, guiding, and explaining progressive health reforms, health justice is distinguished by its embrace of a distinctively *social*, communitarian ethic of care and its reframing of the relationship between health care, public health, and the social determinants of health.^1^ “Rather than merely adopting social justice as the ‘core value’ of public health as … others have done,” health justice demands that social justice should be embraced as “a core value of health law and policy writ large.”^2^ Health justice situates access to health care within a capacious model of the social determinants of health. Rather than describing the social determinants of health as unmet social needs of individuals that exist “upstream” and separate from health care encounters, health justice treats health care access as one among many socially constructed determinants of health outcomes that operate at structural, institutional, and individual levels. Health justice demands a self-critical orientation — that is, health justice requires a probing and critical eye to root out the influence of classism, racism, and other forms of social and cultural bias on the design and implementation of measures purportedly aimed at reducing health disparities. Health justice also requires a commitment to reforms developed through collective action grounded in community engagement, empowerment, and participatory parity.

With regard to the laws governing health care financing and delivery more specifically, a health justice model suggests additional principles.^3^ It recognizes the importance of distinctively collective, *public* interests in universal access to affordable, high quality health care — alongside the individual interests of patients, providers, and payers. It prioritizes prevention and integration of public health goals within health care decisionmaking. Finally, health justice asserts the importance of collective oversight through inclusive, democratic governance — secured through robust protections for the civil and political rights of individuals — to guide stewardship of health care resources and ensure equitable distribution of the burdens and benefits of public investments in health.

As a movement, health justice seeks to recognize and build the power of individuals and communities affected by health inequities to create and sustain conditions that support health and justice.

Emily Benfer’s work on the health justice framework (like that of some of the authors who are featured in this volume) has been particularly influential and is grounded in her work with medical legal partnerships.^4^ Benfer recently collaborated with James Bhandary-Alexander; Yael Cannon, Medha Makhlouf, and Tomar Pierson-Brown to develop an agenda for medical legal partnerships to advance health justice in the post-pandemic world. They organized their agenda around six key themes: (1) transdisciplinary collaboration, (2) upstream interventions, (3) adaptability, (4) racial justice, (5) systemic advocacy, and (6) community-based strategies.^5^ Benfer was initially slated to join us as a co-editor of this issue, but her important work with the Biden administration precluded her from doing so. Elizabeth Tobin Tyler and Joel B. Teitelbaum have published an essential primer of readings in health justice, which has been adopted by many medical legal partnership clinics and other interprofessional academic courses to guide students.^6^

Other scholars have applied, expanded, and refined the health justice framework. Medha Makhlouf has explored the meaning of community in the health justice framework by applying it to the experience of newcomers to the United States.^7^ Angela Harris and Aysha Pamukcu have applied a health justice framework to develop a new civil rights of health.^8^ Matt Lawrence has applied the health justice framework to critique the safety net metaphor for public benefits.^9^ Robyn Powell has applied the health justice framework to address health inequities experienced by people with disabilities.^10^ Thalia González, Alexis Etow, and Cesar De La Vega have applied health justice to school discipline and policing.^11^ Rachel Rebouche has applied the health justice framework to abortion access.^12^

## Health Justice as a Community-Led Movement

Health justice is work that is community-led and seeks to eliminate the poverty, discrimination, and other forms of subordination that have prevented people living in low-income households and communities, Black individuals and members of other racial minority groups, women, disabled people, newcomers to this country, and LGTBQIA+ individuals from accessing resources such as health care, employment, and housing. This work was started by groups such as the Poor People’s Campaign, the Black Panther Party (BPP), the Young Lords, and the AIDS Coalition to Unleash Power (ACT-UP). The BPP and the Young Lords created free health clinics in their neighborhoods that were the foundation for federal qualified health centers, while ACT-UP successfully campaigned for access to HIV and AIDS treatment. The Praxis Project and the Asian Pacific Environmental Network (APEN), who have joined in our discussions about health justice, continue the health justice movement. The Praxis Project, a national movement-support intermediary committed to capacity building for social change, is working on developing fields of work in ways that encourage multi-level, trans-disciplinary learning and collaboration across issues, across the country, and across the globe. APEN’s work is grounded in the leadership of immigrant and refugee community members to develop an alternative agenda for environmental, social and economic justice.

## Health Justice in the Covid-19 Pandemic

The Covid-19 pandemic has provided a particularly compelling and tragic lens through which to understand the value of a health justice approach. Disparities in Covid-19 infections, disease outcomes, and access to healthcare were stark and linked not only to health care system discrimination and inequity, but also to inequity throughout society: certain workers were at greatest risk; residential segregation and lack of employment protections made access to care more difficult for some; many communities lacked the resources needed to help children and families living on the margins to navigate disruptions in education and income. Numerous scholars and practitioners have argued for a more just and effective response to the Covid-19 pandemic (and to the new public health threats already emerging as the perceived threat from Covid-19 subsides). Health justice provides the model for such an approach because it demands legal and policy responses that are structural, supportive, and empowering.^13^

Realizing health justice requires addressing the structural determinants of health that are the root cause of health inequities, such as the social and economic policies that create unequal conditions in health care, employment, housing, and education. This requires attention to the relationship between health care and public health laws and broader patterns of subordination throughout society. Reformers must “address the role of health care laws and policies in reinforcing — or, alternatively, dismantling — racism, economic injustice, and other forms of social subordination.”^14^

Health justice also demands that policymakers and health officials prioritize the provision of material resources and legal protections over interventions aimed at inducing or mandating individual behavior change. This requires an interrogation of inequities in the current distribution of health-related burdens and benefits, and efforts to ensure that on-going public investments in health care and public health are being equitably distributed in accordance with need. In short, health care reformers must prioritize distributive justice — measured with attention to the health outcomes and wellbeing of subordinated communities, in addition to the intermediate indicators of health care access, quality, and cost.

Communities that have been disenfranchised by racism, poverty, and other forms of subordination must be recognized, engaged, respected, and empowered as leaders in the development and implementation of interventions to eliminate health inequities and realize health justice. This means that the processes created to develop, evaluate, and reform laws and policies that shape health must incorporate mechanisms for combatting existing power imbalance and subordination, by centering community decision making and control.^15^

## The Health Justice: Engaging Critical Perspectives in Health Initiative

The mission of the *Health Justice: Engaging Critical Perspectives in Health Law and Policy Initiative*, which we co-chair, is to foster theory, practice, and action on health justice. Our focus is on applying critical perspectives — including critical race theory, Lat Crit, ClassCrit, black feminist theory, feminist legal theory, queer theory, critical disability studies, critical trans legal studies, and more — to the most pressing challenges in health law and policy. We have a big-tent vision of health law and policy, encompassing public health, the social (and structural, legal, economic, and political) determinants of health, health care, bioethics, and global health. We aim to encourage health law and policy scholars, advocates, workers, and justice movement activists to engage more deeply with critical perspectives. We also hope to encourage scholars, advocates, workers, and activists from various critical perspectives who have not previously engaged in the health law and policy sphere to do so as part of this project.

When we came together in 2019 to form an organized initiative to engage critical perspectives in health law and policy to further develop the health justice framework and movement, we recognized that realizing health justice requires building community among academic experts and community organization leaders across disciplines and sectors. We partnered with co-sponsors, including the UCLA Health Law and Policy Program, the Institute for Healing Justice & Equity, ChangeLab Solutions, and the Satcher Health Leadership Institute at Morehouse School of Medicine, which each provided generous financial support for an in-person convening and this open-access journal issue.

Our initial aim was to host a multi-day in-person conference in October 2020 featuring dozens of experts and thought leaders who responded to our call for proposals. When the pandemic hit, we were pulled in multiple directions, but we continued to carve out time for our community-building initiative by hosting a series of virtual workshops that brought together those who responded to our original call for proposals for interactive discussion. These workshops and the October 2020 virtual conference that they fed into (attracting several hundred attendees at a time when we were all still learning the ins and outs of virtual convening) were organized around three key themes: securing distributive justice, valuing human dignity, and empowering communities.

We are grateful to the steering committee that shaped this initiative in its early months for their work on the conceptual framing of these themes: Emily Benfer, Brian Castrucci, Daniel Dawes, Sarah de Guia, Gregg Gonsalves, Nan Hunter, Dayna Bowen Matthew, Jamila Taylor, and — with special gratitude for the many extra hours she devoted to this project — Angela Harris. We also owe a debt to American University’s Health Law and Policy Program, the first home of this initiative, and to the events and information technology offices of American University Washington College of Law (AUWCL), for making the virtual workshops and conference possible. Recordings from the virtual conference are hosted on AUWCL’s Health Law and Policy Program website.

The publication of this symposium marks an important milestone in our efforts to engage critical perspectives in health law and policy, with the goal of facilitating insights that would translate into practical tools for preparing and supporting the next generation of collaborative health leaders. We hope these papers will be useful to teachers in schools of law, public health, nursing, medicine, and public policy. We hope they will inspire new thinking, expansion, and meaning-making within and beyond classrooms and community-led reforms supported by agencies, legislatures, courts, and civic institutions.

In the summer of 2021, we invited some of the participants from our 2020 workshops and conference, as well as new voices who had not previously been part of our initiative, to create a blog symposium hosted by the Petrie Flom Institute’s Bill of Health Blog. Building on this foundation, in the winter of 2021-22, we recruited long-standing participants in this initiative — and new ones — to contribute the papers that appear in this volume and to convene for an in-person workshop hosted by the initiative’s new home, UCLA’s new Health Law and Policy Program, in October 2022.

Our 2022 workshop featured many of the authors of this symposium issue as well as several commentators who enriched the papers that appear in this volume: Devon Carbado, Lauren Clark, Christine Cordero, Daniel Goldberg, Thalia González, Kimberly Libman, Yvonne Mariajiminez, Ilan Meyer, Xavier Morales, and Lauren van Schilfgaarde. We are also grateful to the dozens of anonymous peer reviewers who lent their time and expertise to strengthen the papers we feature here.

Workshop participants expressed many variations on the core themes of respect for community, partnership between scholars and community advocates, and calls to action to achieve health justice. They highlighted the need for social movement organizers and scholars to work together to build more just political, economic, legal, and social systems. They emphasized that doing this work requires coming to terms with the racialized nature of these systems and repairing and redressing the trauma these systems have caused. They focused on the need to ground health justice in the work of building what people need to thrive. Building on other social justice movements, participants emphasized that lawyers and others with specialized expertise should be on call and ready to help, but that community should lead these efforts. Scholars should accompany the community in this journey of equality and equity rather than attempting to take the lead. Some participants pointed out that this commitment to community-led efforts must involve granting communities the right to make mistakes as they try to address historical and community traumas. A central theme of the workshop was a call to break down silos and work collaboratively with community organizations and scholars in areas beyond those traditionally understood as being within the purview of health law and policy, such as education, the criminal legal system, the immigration system, labor and employment law, housing, transportation, food justice, and political economy.

Throughout these efforts we have developed a growing bibliography of resources for advocates, authors, and commentators to use as they see fit, but we resisted defining health justice for the authors whose work is featured in this issue. We view health justice as an open-weave framework, which invites expansion, refinement, and meaning-making from multiple perspectives.

## Next Steps

Our initiative is ongoing. The publication of this symposium marks an important milestone in our efforts to engage critical perspectives in health law and policy, with the goal of facilitating insights that would translate into practical tools for preparing and supporting the next generation of collaborative health leaders. We hope these papers will be useful to teachers in schools of law, public health, nursing, medicine, and public policy. We hope they will inspire new thinking, expansion, and meaning-making within and beyond classrooms and community-led reforms supported by agencies, legislatures, courts, and civic institutions. We are honored to provide this platform for the work of an immensely talented group of authors and grateful for the friendships, fellowship, community, and ongoing collaborations this project has fostered.
